# A molecular perspective of gelsolin amyloidosis: An old foe with new faces

**DOI:** 10.1007/s00018-026-06172-7

**Published:** 2026-03-15

**Authors:** Michela Bollati, Carmina Natale, Loic Girois, Andrea Conz, Kaliroi Peqini, Stefano Pieraccini, Sara Pellegrino, Luisa Diomede, Matteo de Rosa

**Affiliations:** 1 Institute of Biophysics, National Research Council, Via Alfonso Corti 12, 20133 Milano, Italy; 2 Department of Molecular Biochemistry and Pharmacology, Istituto Di Ricerche Farmacologiche Mario Negri IRCCS, Via Mario Negri 2, 20156 Milano, Italy; 3 Department of Pharmaceutical Science, University of Milano, Via Golgi 19, 20133 Milano, Italy; 4 Department of Chemistry, University of Milano, Via Golgi 19, 20133 Milano, Italy

**Keywords:** Amyloidosis, Gelsolin, Protein structure, Molecular biophysics, Aggregation, Drug discovery

## Abstract

Hereditary gelsolin amyloidosis (AGel) is a rare and often underrecognized protein misfolding disorder caused by mutations in the gelsolin (GSN) protein, leading to its aggregation in various tissues. Its rarity, combined with a heterogeneous and complex clinical presentation and the multidomain, flexible nature of GSN, has impeded research into its pathogenic mechanisms and therapeutic options. GSN comprises six homologous domains, labeled sequentially from G1 to G6. For over 40 years, AGel amyloidosis was exclusively linked to a systemic form, caused by D187N and D187Y mutations in the second domain, referred to as the Finnish and Danish variants. However, since 2013, numerous novel amyloidogenic variants have been identified in different protein regions, leading to various clinical phenotypes, characterized by distinct molecular mechanisms. This review examines these mutations and proposes a classification based on molecular and clinical features to enhance research and diagnosis. Additionally, we summarize whether elucidating the different pathogenic mechanisms aids in identifying potential druggable targets. The lack of information and biological models and limited efforts to develop pharmacological treatments highlight the need for further therapeutic exploration.

## Introduction to systemic and localized amyloidoses of hereditary or sporadic origin

Amyloidoses, also known as protein misfolding disorders, are a heterogeneous group of diseases caused by endogenous extracellular or intracellular proteins that can undergo a conformational change from an unstable native state to an alternative one characterized by antiparallel β-sheets. This conformational rearrangement stabilizes the protein, allowing it to form soluble oligomeric structures and insoluble fibrillar complexes that can deposit and accumulate in various tissues and/or organs, compromising their functionality [1, 2]. These fibrillar aggregates are commonly referred to as amyloidogenic deposits. The term ‘amyloid’ is derived from the Latin '*amyloideus*' and was first used in 1854 by Rudolf Virchow to describe a small protein deposit with starch-like properties.

The classification of diseases caused by amyloidogenic proteins is based on the biochemical nature of the protein precursor and the proteins responsible for generating deposits, thus defining the pathology [3]. To date, 42 human amyloidogenic proteins have been identified, each associated with a specific disease [4, 5]. All these proteins can adopt a β-fibrillar structure and produce insoluble deposits composed of amyloid fibers with diameters ranging from 7 to 13 nm and variable lengths. Non-fibrillar constituents are also present in the deposits, including glycosaminoglycans, serum amyloid protein, and, to a lesser extent, apolipoprotein E, type IV or VI collagen, and laminin [6]. Amyloidogenic proteins are categorized according to their origin and functional or pathological role. Some proteins form disease-associated amyloid deposits, such as immunoglobulin light chains in primary amyloidosis, transthyretin in hereditary and wild-type (WT) forms, and serum amyloid protein A in chronic inflammatory conditions [5]. However, other proteins are involved in the formation of functional fibrils with specific physiological roles, such as the major basic protein of eosinophilic granulocytes, the melanosome protein PMEL-17 in melanosome formation, and bacterial amyloid proteins in biofilms [5]. Regardless of the protein that forms them, all amyloidogenic deposits are characterized by specific biochemical properties, such as resistance to denaturing agents and proteolytic degradation [7]. Furthermore, fibrils have a distinctive fibrillar-type ultrastructure that appears highly ordered under the electron microscope and displays specific X-ray crystallographic diffraction patterns [8]. Dyes like Congo Red and thioflavin T (ThT) specifically bind to this structure [7, 9], producing a characteristic birefringence [10].

**Fig. 1:**
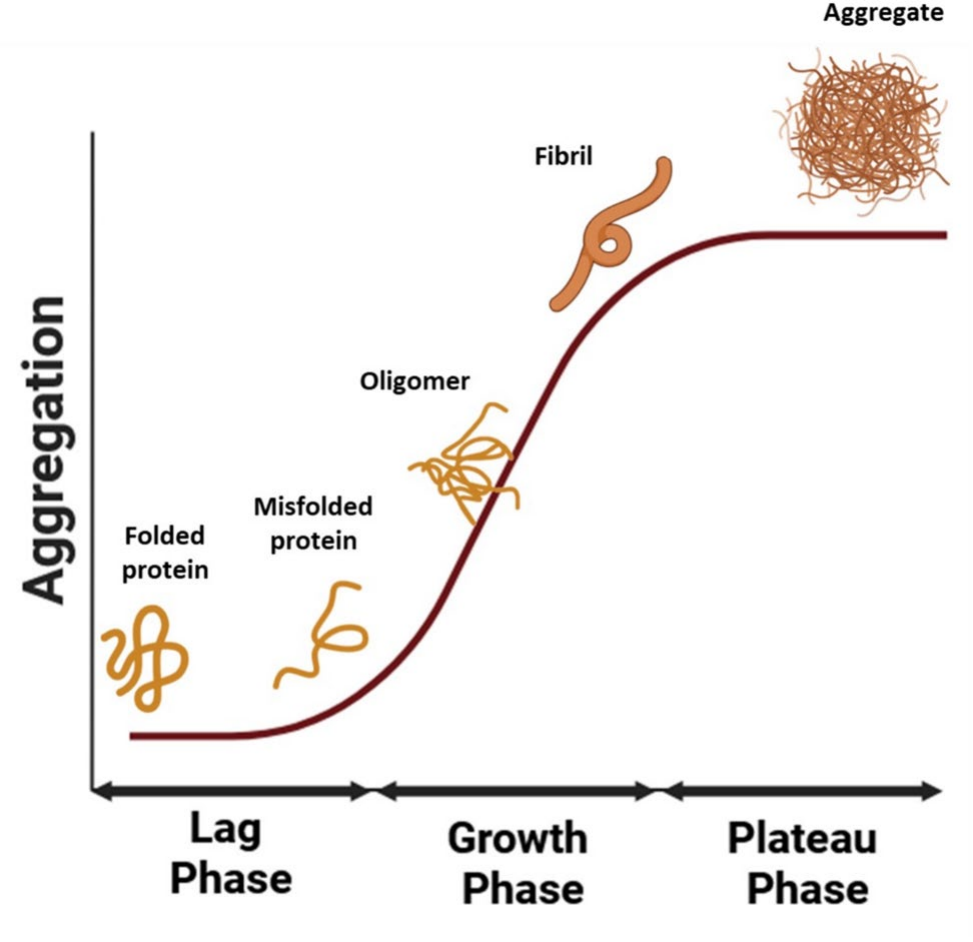
Molecular events underlying amyloid aggregation. All amyloidoses stem from a misfolding event that eludes chaperone-assisted refolding and degradation. The misfolded protein exposes aggregation-prone regions that drive the formation of nuclei of aggregation, yet soluble oligomers already characterized by a b-structure. Oligomers grow into longer fibers that are eventually deposited and decorated by other proteins characterizing amyloid aggregates. The kinetic of amyloid aggregation is characterized by a first lag phase due to the formation of nuclei, followed by a growth phase where elongation and secondary nucleation occur. Eventually a steady state is reached. This schematic representation is intentionally simplified; for a comprehensive discussion of the molecular complexity of amyloid-formation pathways, readers are referred to dedicated reviews [12]

The mechanism underlying protein aggregation was first described for the amyloid β (Aβ) protein, whose accumulation in the brain is considered the molecular driver of the onset and progression of Alzheimer’s disease. It was later shown that this process, termed the ‘amyloid cascade’, can also be generalized to other amyloidogenic proteins, suggesting that the formation of proteotoxic species from a precursor protein and their aggregation are not specific to a particular protein [11]. Amyloid aggregation is a complex process characterized by equilibria among multiple transient species and competing pathways, as comprehensively reviewed here [12]. In a much-simplified view, during the early stages of the aggregation process, the monomers of the amyloidogenic protein begin to self-assemble, forming small, soluble, and unstable intermediates known as oligomers (Fig. 1). Hydrogen bonds can form between amino and carbonyl groups within these soluble intermediates, leading to the generation of chains called protofibrils [4, 13], which can subsequently assemble longitudinally into mature fibrils, creating the amyloidogenic deposits found in patients suffering from amyloidosis [13, 14].

Whether oligomers, amyloid protofibrils, or fibrils are responsible for the onset and progression of the disease remains a matter of debate [15]. The fibrillar deposits were considered the most toxic species driving tissue and organ dysfunction for a long time. Both pre-clinical and clinical evidence indicate that while the accumulation of aggregates in extra or intracellular compartments alters tissue structure, the soluble oligomers, due to the exposure of their hydrophobic domains, are responsible for the proteotoxicity [4, 13, 16, 17].

Based on the organs where fibrillar aggregates are deposited in the human body, amyloidoses can be classified as localized or systemic [5, 18]. Localized amyloidosis remains confined to a specific area, while systemic amyloidosis involves a broader spread. Among the human fibrillar amyloid proteins, 19 are associated with systemic deposits, while 4 occur with either localized or systemic deposits [5].

Among localized amyloidoses, central amyloidosis is the most common form, characterized by deposits in the central nervous system, which is also where the amyloidogenic precursor protein is produced. This type of amyloidosis encompasses several neurodegenerative diseases, including Alzheimer's disease, which is a double proteinopathy marked by deposits of Aβ and hyperphosphorylated Tau; Parkinson's disease, associated with α-synuclein aggregation; and Huntington’s disease, linked to polyQ-expanded huntingtin deposits [19]. In systemic amyloidoses, the protein precursor is transported from the synthesis site through the bloodstream into the extracellular compartments of various organs and/or tissues, leading to the formation of amyloid deposits. These disorders are rare, with immunoglobulin light chain amyloidosis and transthyretin amyloidosis (ATTR amyloidosis) having the highest incidence in Western countries [20]. Approximately 20 proteins have been identified as responsible for systemic amyloidosis in humans. Although they differ significantly in structure and function, they all produce morphologically indistinguishable aggregates [21].

Amyloidoses can be further divided into hereditary and sporadic forms, with the latter accounting for approximately 95%. Hereditary localized and systemic amyloidoses are characterized by autosomal dominant mutations that result in the formation of mutant proteins with a strong tendency to aggregate. This is relevant to familial Alzheimer’s disease, which arises from mutations in amyloid precursor protein and Aβ, as well as in hereditary systemic amyloidosis, which is highlighted by variants of apolipoprotein AI, apolipoprotein AII, lysozyme, fibrinogen A chain α, gelsolin (GSN) [6], and notably transthyretin with more than 130 different inherited genetic variants [22].

Among systemic amyloidoses, hereditary gelsolin amyloidosis (AGel) is of scientific and clinical interest because it is caused by specific mutations in the *gsn* gene, which lead to the aggregation and deposition of protein fragments. Clinical presentation is heterogeneous and often includes peripheral neuropathy, muscle weakness, and cardiac or renal involvement [23, 24].

Diagnosing AGel is a multifaceted process typically involving genetic testing, tissue biopsy, and advanced imaging techniques. The GSN mutations associated with AGel can be identified through next-generation sequencing. Histologic and histochemical analysis paired with Congo Red staining and electron microscopy are traditionally used to reveal the characteristic apple-green birefringence and the presence of fibrils [10, 25]. Imaging techniques, such as echocardiography or magnetic resonance imaging, may assist in assessing organ involvement, particularly in cases where cardiac amyloidosis is suspected [26]. A breakthrough in amyloidosis diagnosis has emerged from laser microdissection followed by mass spectrometry analysis of Congo Red-positive areas [27–29]. This method also allows for the typing of amyloidosis by identifying the causative protein. Early diagnosis through these approaches is vital for effective management, as amyloid deposits can lead to progressive organ dysfunction if left untreated.

## Introduction to GSN structure, biochemistry and physiological activities

GSN is a calcium-dependent regulator of actin filament dynamics. The 70-kb *gsn* gene, located on chromosome 9, was first sequenced by Kwiatkowski and colleagues in 1986 [30, 31]. GSN is a well characterized and almost ubiquitous actin-binding protein, playing a central role in cytoskeletal remodeling during cellular processes such as differentiation, movement, and apoptosis [32]. Like other proteins in the same superfamily, GSN can sever actin filaments while remaining attached to the resulting barbed (plus) ends, where polymerization is favored. In this way, GSN prevents filament growth and creates more pointed (minus) ends, facilitating depolymerization [33, 34]. Given these key activities, GSN is involved in numerous biological processes such as cell motility and division, signal transduction, apoptosis, transcriptional coactivation, and consequently in various pathological conditions. These aspects of GSN have been extensively reviewed elsewhere [35, 36].

Three isoforms of the GSN protein are expressed from the *gsn* gene. The cytosolic form is an 80 kDa protein composed of 730 amino acids, while the plasma GSN is an 83 kDa secreted isoform [34]. The secreted form is thought to sever free actin filaments in plasma, which helps reduce viscosity and regulate inflammation [31]. Plasma GSN undergoes cleavage of its N-terminal 27-residue signal peptide, enabling secretion through the endoplasmic reticulum. The mature plasma form is used as a reference for residue numbering throughout this text. The third isoform, GSN-3, is the least characterized and appears to be specific to the central nervous system, with an N-terminus that is 11 amino acids longer than the cytoplasmic protein [37].

**Fig. 2:**
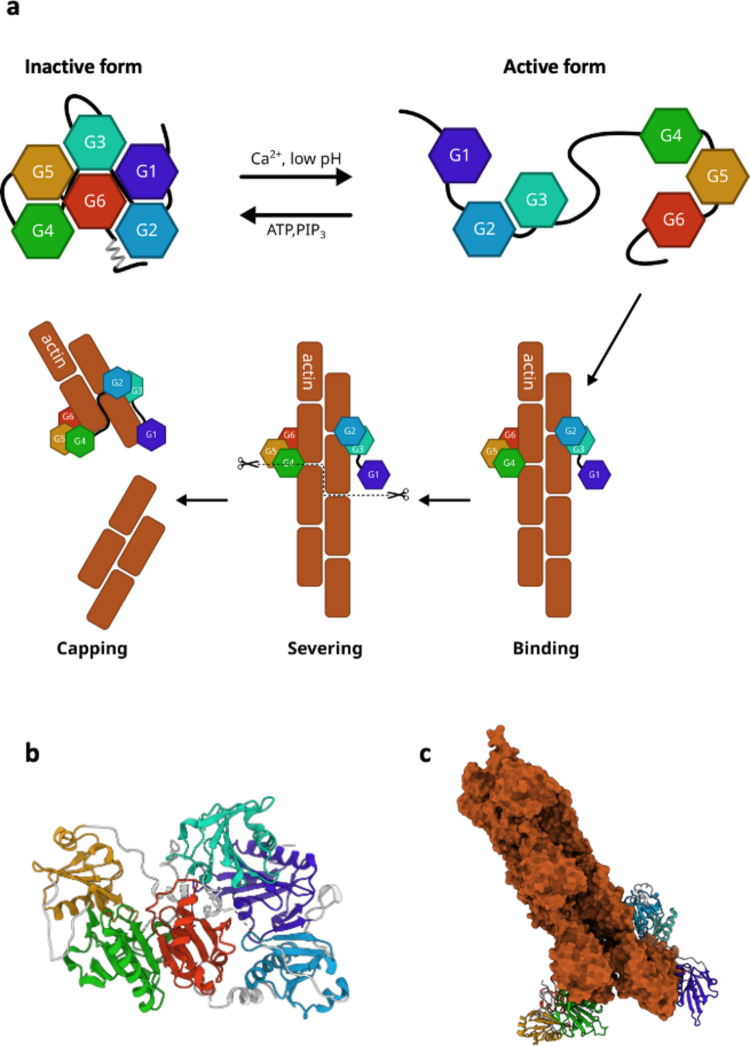
GSN structure and conformational changes required to bind, sever, and cap filamentous actin. **a** In the absence of calcium ions, GSN adopts a compact conformation called the inactive form. Activation is achieved by opening the structure, which can be induced by Ca^2+^, low pH, and temperature. Opening is reverted by binding to ATP or inositol phosphates (PIPs). In the open/active conformation, GSN can bind actin filaments (f-actin), promoting its severing. GSN can also bind the plus (+) end of the filament (capping activity) and prevent polymerization. GSN can potentially adopt at least four different conformations; however, atomic models are available only for the b closed/inactive and c the capping states

All GSN isoforms consist of six homologous domains, labeled sequentially from G1 to G6 (Fig. 2). This modular structure arises from two successive gene duplications of the core GSN domain, followed by duplication of the resulting triplet. Consequently, the domains form pairs exhibiting high sequence and structural similarity: G1 and G4, G2 and G5, and G3 and G6 [38, 39]. All homologous domains share a GSN-like fold, comprising a five-stranded β-sheet flanked by two α-helices. GSN contains eight calcium-binding sites: one type-2 site in each domain and two additional type-1 sites, in G1 and G4, that facilitate actin binding [38, 40, 41].

GSN activity is regulated by various molecules and environmental factors, including Ca^2+^, phosphatidylinositol phosphates (PIPs), and pH [42, 43] (Fig. 2). Ca^2+^ is the primary and most well-characterized modulator, promoting a concentration-dependent activation that allows GSN to bind to actin filaments. In contrast, PIPs and adenosine triphosphate (ATP) inhibit the GSN-actin interaction, releasing GSN from the barbed ends when capping is no longer necessary, and filament elongation is desired [44–46].

GSN adopts a compact, self-inhibited conformation in a calcium-free environment with a tightly packed structure. Numerous crystal structures of the full-length WT and mutated GSN have been resolved over the years [38, 47–49] (Table 1), revealing G1 to G5 wrapped around G6, with the C-terminal latch binding to the G2 domain to lock the protein in this inactive state. The type-1 sites are buried in this configuration, rendering them inaccessible for actin binding.

**Table 1:** Summary of the experimental structures of human GSN

Construct	PBD code	Notes
Full-length		
GSN WT	3FFN	close/inactive conformation [38]
	8VIZ	Ca^2+^-bound in complex with f-actin (capping) [58]
GSN D187Y	6JEH	amyloidogenic mutation, close/inactive conformation [48]
GSN D187N	6JCO, 6QBF	amyloidogenic mutation, close/inactive conformation [48, 49]
GSN G167R	6JEG, 6Q9Z	amyloidogenic mutation, close/inactive conformation [48, 49]
GSN N184K	6Q9R	amyloidogenic mutation, close/inactive conformation [49]
GSN A551P	7P2B	amyloidogenic mutation, close/inactive conformation [47]
Isolated domains		
G1	5ZZ0	Ca^2+^-bound [143]
	1P8Z, 1C0F, 1C0G, 1D4X	Ca^2+^-bound in complex with g-actin [144]
	1MDU	Ca^2+^-bound in complex with f-actin [145]
	5UBO	in complex with Mical-oxidised g-actin [146]
G2 WT	1KCQ, 6QW3	Cd^2+^ and Ca^2+^-bound [72, 73]
G2 WT	4S10	Ca^2+^-bound in complex with Nb11 [124]
G2 D187N	6H1F	amyloidogenic mutation, in complex with Nb11 [71]
G2 N184K	5FAE, 5FAF	amyloidogenic mutation [78] Ca^2+^-bound
G2 G167R	5O2Z	amyloidogenic mutation, as a domain-swap dimer [57] Ca^2+^-bound
G3	6LJE, 6LJF	Ca^2+^-bound [147]
Triplets		
G1-G3	3FFK	Ca^2+^-bound in complex with g-actin [38]
	8VKH	Ca^2+^-bound in complex with f-actin (capping) [58]
G4-G6	2FH1, 2FH2, 2FH3, 2FH4, 1P8X	Ca^2+^-bound and free [148, 149]
	1H1V	Ca^2+^-bound in complex with f-actin [41]

*Nb11* nanobody 11

GSN senses Ca^2+^ across a broad concentration range, likely due to the individual affinities of its domains, which range from 0.2 to 600 µM [41, 50–52]. Small-angle X-ray scattering (SAXS) and radiolytic protein footprinting indicate that upon ion binding, the packing of the domains relaxes, resulting in an increased hydrodynamic radius of GSN and changes in surface accessibility, leading to its fully active form [52, 53]. Research on other members of the GSN superfamily suggests that the active/open conformation likely exists as an ensemble of conformations, with the six domains no longer constrained by dynamic equilibrium [54]. Due to its flexible conformation, Ca^2+^-bound active GSN has evaded high-resolution structural characterization, and studies have concentrated on the isolated domains or triplets in the presence of the ion. A comprehensive list of available GSN experimental structures can be found in Table 1.

The triplets G1-G3 and G4-G6 exist in vivo as a product of caspase 3 [55, 56]. This is a Ca^2+^-independent and irreversible activation of GSN, the fragments participate as effectors in apoptotic processes, by severing actin filaments, proteolyzed GSN causes cell rounding, detachment, and nuclear fragmentation. The crystallographic structures of the two halves of GSN, specifically G1-G3 and G4-G6, bound to actin are available [38, 41]. Both halves of GSN unwrap as the third domain releases the first domain, shifting aside to reveal the actin-binding regions on domains G1 and G4.

GSN activation is detectable in vitro using various structural and biophysical techniques, such as SAXS, radiolytic protein footprinting, and thermal denaturation assays. SAXS and radiolytic protein footprinting experiments allow for the observation of the increase in GSN's hydrodynamic radius and changes in surface accessibility, respectively, due to the relaxation and opening of the structure upon the addition of Ca^2+^ [52, 53]. Conversely, pressure and temperature denaturation assays monitor structural melting by examining the different behaviors in the presence or absence of Ca^2+^ [47, 49, 57]. The compact Ca^2+^-free form resembles a globular protein characterized by a single transition melting curve between native and denatured states. In the presence of calcium, GSN unwinds, causing the domains to behave independently, which results in a slanting multistep profile.

Recently, cryo-electron microscopy (cryo-EM) has resolved the structure of the GSN:actin capping complex [58] (Fig. 2). This structure once again demonstrates the intrinsic plasticity of GSN, as it can wrap around the actin filament by extending the long G3-G4 linker.

## GSN mutations and mechanisms underlying AGel amyloidosis

**Fig. 3:**
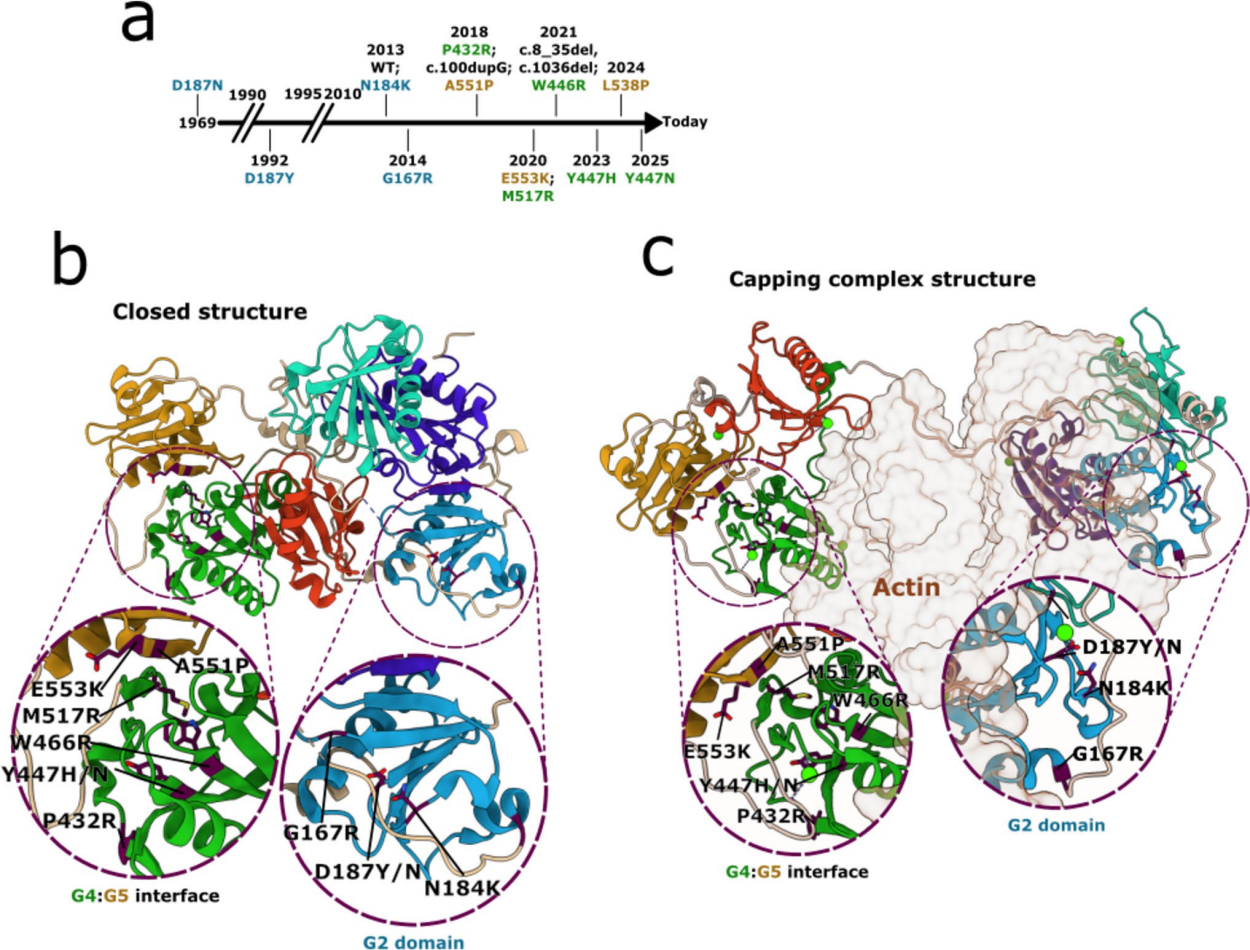
**a** The Timeline for identifying novel pathogenic mutations is color-coded according to the hosting domain. Crystallographic structure of GSN in the **b** closed/inactive conformation and **c** cryo-EM structure of the active form in complex with f-actin. In the inset is a close-up view of the two regions harboring most of the amyloidogenic mutations, the G2 domain and the G4:G5 interface

AGel amyloidosis was first described in Finland in 1969 [59] and consequently named familial amyloidosis Finnish-type (FAF). For a long time, the disease was associated only with two substitutions of the same residue (D187N/Y) and was believed to be geographically restricted to endemic areas in northern Europe. AGel amyloidosis is very rare, and its clinical presentation is complex and heterogeneous, likely remaining undiagnosed for years. Recently, there has been an increased awareness of this class of disease, broader use of genetic tests, and advancements in diagnostic tools [29] that have shown AGel amyloidosis is present worldwide, even in families without Finnish ancestors [60–62]. Since 2013, many novel mutations have been identified (Fig. 3 and Table 2) as responsible for the disease, which is sometimes characterized by an alternative presentation of symptoms. All single-point mutations causing AGel amyloidosis lead to a gain-of-toxic function phenotype, resulting from the extracellular deposition of GSN protein in an insoluble beta-pleated sheet conformation.

**Table 2:** Identification and clinical features of GSN amyloidogenic variants

Variant	Substitution	Domain	Phenotype	First description
D187N	c.654G > A (p.D214N)	G2 (Ca binding site)	Systemic (cranial neuropathy and peripheral sensory polyneuropathy, corneal dystrophy, *cutis laxa*), renal and heart complications at times	*1969 *[59]
D187Y	c.654G > T (p.D214Y)	G2 (Ca binding site)	Systemic (cranial and peripheral neuropathy, corneal dystrophy, *cutis laxa*), renal and heart complications at times	*1992 *[63]
N184K	c.633C > A (p.N211K)	G2	Renal localized	*2013 *[81]
G167R	c.580G > A (p.G194R)	G2	Renal localized	*2014 *[80]
A551P	c.1732G > C (p.A578P)	G5 (G4: G5 interface)	Patient also affected by ATTR amyloidosis. No evidence of overlapping tissue involvement by the 2 types, with GSN deposits in fat aspirate and descending colon and ATTRv in the heart. No clinical signs typical of AGel	*2018 *[84]
E553K	c.1738G > A (p.E580K)	G5 (G4: G5 interface)	*Cutis laxa*, cranial neuropathy, including the optic nerve, and cardiac involvement	*2021 *[87]
M517R	c.1631 T > G (p.M544R)	G4 (G4: G5 interface)	Corneal lattice dystrophy, *cutis laxa*, and facultative peripheral neuropathy	*2020 *[132]
W466R	c.1477 T > C (p.W493R)	G4	Polymorphic corneal stromal opacities and other systemic ophthalmic features	*2021 *[88]
Y447H	c.1420 T > C (p.Y474H)	G4	Mild bilateral carpal tunnel syndrome. Amyloid deposits in breast tissue	*2023 *[85]
Y447N	c.1420 T > A (p.Y474N)	G4	Patient also affected by WT ATTR amyloidosis. Cardiac amyloidosis, GSN amyloid in the heart vasculature, ATTR in the cardiac interstitium. Carpal tunnel syndrome	*2025 *[86]
P432R	c.1375C > G (p.P459R)	G4 (G3: G4 interface)	Systemic atypical (fevers, skin rash, polyneuropathy, and anemia)	*2018 *[95]
L538P	c.1613 T > C (p.L511P)	G5 (G4:G5 interface)	Retinal corneal dystrophy,Carpal tunnel syndrome. Deposits in both the stroma and vascular walls	*2024 *[96]
WT	-	-	Amyloid deposition in pituicytoma or tumor-associated Agel	*2013 *[97]
c.100dupG	pA34fs	G1	Seizures, multiple cerebral cavernous malformations, and haemorrhagic brain lesions	*2018 *[90]
c.8_35 del	p.P3fs	G1	Cognitive dysfunction, mild peripheral neurological symptoms, and no eye or skin symptoms	*2020* [91]
c.1036delA	p.K346fs	G3	Cognitive dysfunction, personality changes, psychiatric symptoms, and symptoms in multiple body systems	*2020* [91]

By reviewing the literature, we here propose a tentative classification of the known amyloidogenic mutations based on a combination of clinical and molecular features of AGel amyloidosis: i) a systemic form, caused by D187N and D187Y mutations in the second domain, respectively known as the Finnish- and Danish-variant; ii) mutations that although hosted by the G2 domain cause a kidney localized form of the disease; iii) a sporadic form, caused by WT GSN deposits surrounding a sellar glioma of the hypophysis; iv) variants carrying mutations at the interface between domains G4:G5; and v) frame-shift mutations responsible for an Alzheimer’s disease-like phenotype.

### The systemic Finnish and Danish forms

The first identified and better characterized AGel mutations are D187N/Y (numbering according to the mature plasma protein), hosted by the G2 domain [59, 63] (Table 2). In these patients, amyloid fibrils are systemically deposited in the walls of blood vessels and perineural sheaths, giving rise to a slowly progressive neurological deterioration mainly characterized by a triad of clinical manifestations: corneal lattice dystrophy, *cutis laxa*, and sensory polyneuropathy [23]. These manifestations are caused by the abnormal deposition of GSN amyloid fragments in various tissues. The earliest symptom is typically corneal lattice dystrophy in the third or fourth decades of life [64], which occurs when GSN amyloid accumulates in the peripheral cornea in a lace-like pattern, as well as in the associated nerves. Amyloid deposits along large nerve fibers lead to neurological involvement, which generally appears later in the disease, initially affecting the facial nerves, followed by involvement of other cranial nerves such as trigeminal, glossopharyngeal, and hypoglossal nerves. Patients often present with a combination of symptoms, including facial nerve palsy, mild polyneuropathy, hearing loss, ataxia, dysarthria, and orthostatic hypotension [65]. As the disease progresses, *cutis laxa* develops. This condition results from the deposition of GSN amyloid in the basement membrane of the dermis, which disrupts the dermal architecture and impairs the normal function of collagen and elastin fibers. Nearly all organs are affected to some extent. In fact, although AGel amyloidosis predominantly involves the nerves, amyloid deposits have been found in 80% of organs, primarily in the heart, kidney, soft tissue (*i.e.,* carpal tunnel syndrome and breast masses are common), lung, liver, and pancreas, confirming AGel as a systemic disease. Despite the progression of the disease and organ involvement, individuals with AGel amyloidosis typically have a normal life expectancy [24].

AGel patients' tissues from the peripheral and central nervous systems [66] contain amyloid fibrils composed of a 71-residue fragment of GSN, specifically amino acids 173–243 [67]. Analysis of the D187N/Y amyloidogenic variants revealed an aberrant proteolytic cleavage at position R172|A173 of G2, usually covered by the G3 domain, by an “alpha-gelsolinase”, later identified as the furin protease [68]. Furin produces a 68 kDa C-terminal fragment (C68), which is secreted in the extracellular space where it undergoes a second cleavage, giving the amyloidogenic peptides [69, 70].

Biophysical characterization of the variants showed that D187N/Y substitutions do not induce large changes in the calcium-free full-length GSN structure and do not affect its physiological activity [48, 49]. The thermal stability of the mutated full-length proteins is almost similar to that of the WT (Table 3), suggesting that the mutated full-length protein is as stable as the WT both in the presence and in the absence of calcium. When tested for their propensity to aggregate using thioflavin T, a fluorogenic dye specific for the detection of amyloid-like structures, D187N/Y full-length do not show any propensity to aggregate [48, 49]. When tested in furin proteolysis assays, the mutated proteins show an increased susceptibility to the cleavage [48, 49].

**Table 3:** Molecular features of novel pathogenic mutants. The table includes all variants that have been characterized as recombinant proteins. A comparative value to WT between – and + + was assigned, depending on whether the mutation reduces or enhances that particular feature. Different experimental techniques are used under conditions that are not necessarily identical; as such, the table should only be used as a general reference. The molecular features reported include: susceptibility to furin cleavage, ability to bind calcium, stability measured on the isolated domain and the full-length (FL) protein, and whether the mutation impacts one or both forms of the protein, conformational flexibility, aggregation propensity of the full length protein, proteotoxicity, and which peptides are found in the deposits of the patients

Variant	Furin	Ca^2+^ binding	Destabilization			Flexibility	Aggregation	Toxicity	Deposits
			Domain	FL	Diffusion				
D187N	+ +	-	+ + (G2)	=	Local	+ (G2-G3)	=	+	5/8 kDa fragments
D187Y	+ +	-	+ + (G2)	=	Local	+ (G2-G3)	=	na	5/8 kDa fragments
N184K	+ +	=	+ + (G2)	=	Local	na	na	+	na
G167R	+ +	=	+ (G2)	+	Local/global	+ + (G2-G3)	na	+	5/8 kDa, and non-canonical fragments
A551P	=	=	=/+ (G5)	+	Global	+ (G4-G5)	+	+	Non-canonical fragments
E553K	=	=	+ + (G5)	+ +	Local/global	+ + (G4-G5)	+ +	+ +	na
M517R	=	=	na	+	Local/global	+ + (G4-G5)	+ +	+ +	na

*na* not available

Studies on the isolated G2 domains revealed more biophysical insights. Structural investigation of the molecular causes of the aberrant cleavage shows that D187 belongs to the cluster of residues involved in the chelation of the calcium ion and substitution to N and Y prevents Ca^2+^-binding to the domain [68, 71, 72]. In G2, calcium is hexacoordinated by G186, D187, E209 and D259 residues and two water molecules. D187 contributes one carboxyl oxygen to the binding of Ca^2+^, whereas the other carboxyl-oxygen forms a hydrogen bond with the side chain of K166 [72, 73]. Substitutions of D187 to N or Y cause a loss of the ability to form charge–charge interactions or potential hydrogen bonds with partner residues Q164, K166, and N184 in the core of G2 cluster.

**Fig. 4:**
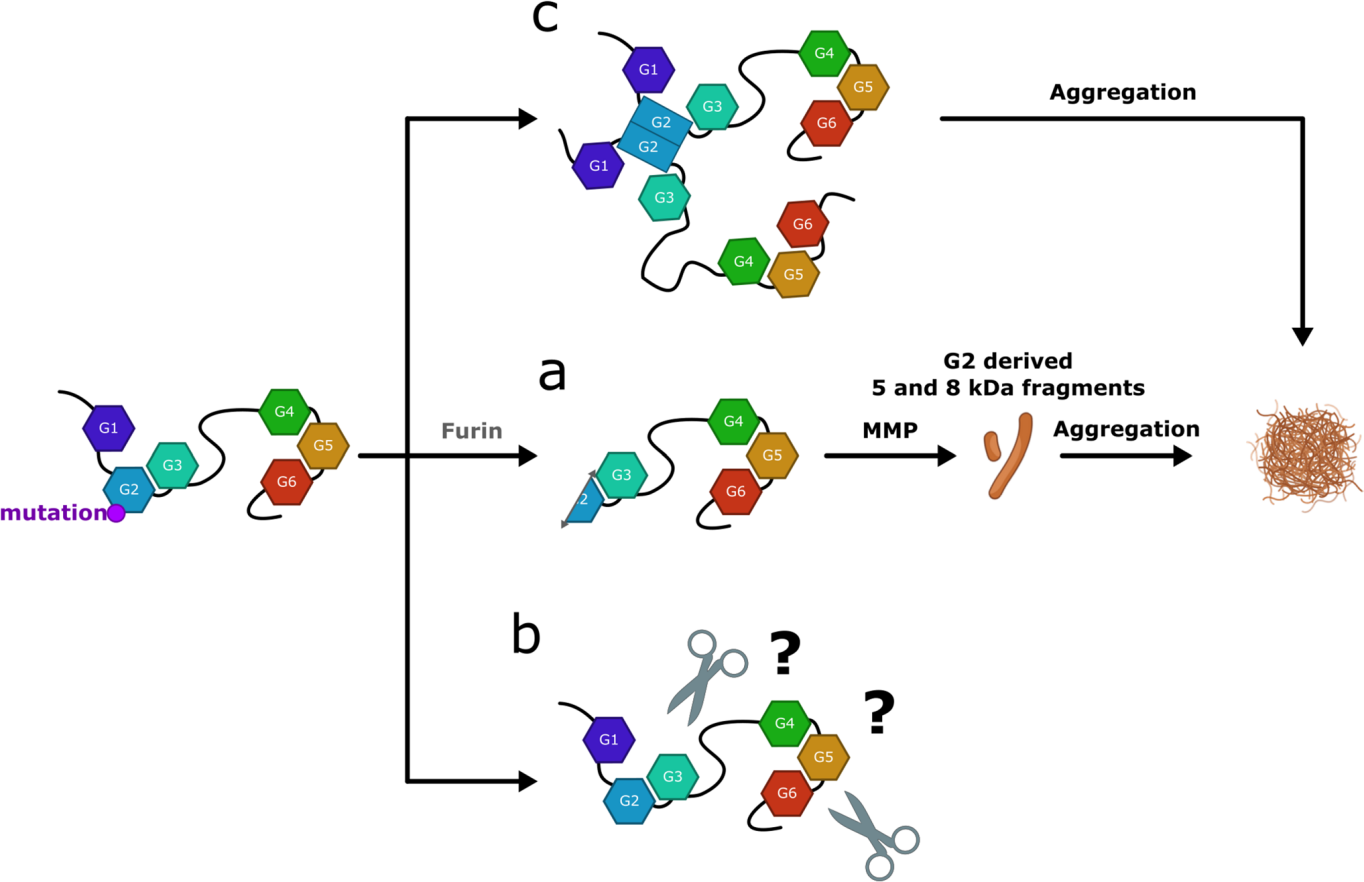
Possible molecular events leading to the deposition of aggregated GSN. Only for the D187N/Y GSN variants the underlying mechanism is well defined and is characterized by two proteolytic events. This furin pathway **a**, named after the first proteases, is also considered the classic one and might be shared by all G2 mutants. Alternative pathways may include b yet-to-be-identified proteases or c proteolysis-independent oligomerization of the protein in a native-like conformation

According to the proposed mechanism (Fig. 4), the impairment of the physiological conformational changes during the calcium activation in the Golgi apparatus cause an increased destabilization of the second domain, as suggested by the strong destabilization of the protein measured in both chemical and thermal denaturation studies [71, 74, 75]. The consequent increased conformational flexibility causes the exposure of a furin protease cleavage site (169-RVVR↓A-173), which is then targeted during secretion in the trans-Golgi environment. In addition, the D187N/Y mutation also disrupts the interaction between G2 and G3 [48]. Domain G3 sterically protects the furin cleavage site, and a FAF-like molecular phenotype can be obtained by mutating this domain. The cleavage product is named C68, a truncated C-terminal protease-sensitive 68 kDa portion that, when transferred to the extracellular matrix, is further processed by matrix metalloproteases, eventually producing the 8 and 5 kDa fragments, composed of the residues A173-M243 and A173-R225, respectively. These macromolecules are amyloid-prone as they possess a highly amyloidogenic sequence spanning residues 182 to 192 [76] or 187 to 193 [77]. The isolated G2 domain itself also has a strong tendency to aggregate [78, 79], while the WT GSN in the full-length form has a weak tendency to aggregate under physiological conditions [47, 75].

### Novel G2 variants with kidney-localized deposition

The first two AGel variants identified, which do not involve D187 substitutions, are N184K and G167R [80, 81] (Table 2). Patients with these substitutions exhibit a rare form of renal amyloidosis, characterized by severe kidney complications that may progress to end-stage renal disease. This condition results from the deposition of GSN amyloid in the glomeruli [82]. No additional clinical features typical of Agel amyloidosis were observed. Although both residues do not directly coordinate calcium, N184 stabilizes the cluster through hydrogen bonds with G186 and D187. Notably, G167 does not directly interact with the cluster residues. Both substitutions maintain the ability to bind Ca^2+^ and preserve the overall cluster geometry, challenging the previous dogma. Like D187N/Y, the thermodynamic stability and three-dimensional structures of the full-length N184K protein are only slightly impacted by the substitutions [48, 49]. In the isolated G2 domain context, the N184K variant displays an even greater local thermodynamic destabilization, leading to increased conformational flexibility [78]. Regarding G167R, the larger arginine side chain disrupts the Ca^2+^ binding cluster by destabilizing the D259 residue and the main-chain conformation, enhancing flexibility of the G2-G3 domains [48, 49]. In both instances, such destabilization triggers the same furin-dependent proteolytic cascade, resulting in the deposition of 8 and 5 kDa amyloidogenic fragments. Interestingly, the G167R substitution has also been shown to promote the dimerization of the G2 domain through a unique domain swap mechanism, where the β1-strand of another monomer replaces the N-terminal β1-strand of one monomer [57]. Dimerization was also observed for the full-length G167R protein; however, high-resolution structural data are not yet available to confirm the nature of this assembly. As a possible consequence, the full-length G167R protein is less stable than the other G2 substitutions. In systemic AGel caused by D187Y/N, the disease arises solely from the exported isoform of the protein, while the full-length mutant protein is not intrinsically amyloidogenic. Indeed, only the 5 kDa and 8 kDa fragments are found in fibrillar tangles [76, 83]. Laser microdissection and mass spectrometry analysis of deposits in patients carrying the G167R substitution reveal GSN fragments that do not correspond exclusively to the canonical 5 kDa and 8 kDa fragments [80]. This may indicate that the full-length protein could also aggregate through the domain swap mechanism. Both N184K and G167R mutations have been shown to increase GSN susceptibility to furin proteolysis [48, 57, 78], suggesting that the same proteolytic pathway described for D187N/Y may be responsible for the amyloid deposition occurring in patients affected by these variants.

The reason why these variants lead to localized, rather than systemic, amyloid deposition remains unclear. Under comparable conditions, the N184K mutation exhibits a lower aggregation propensity than D187N [78], suggesting that higher local protein concentrations—such as those found in the kidney—may be necessary to trigger deposition. The G167R variant is cleaved by furin after A172, generating 5 and 8 kDa fragments similar to the WT, which are presumably less amyloidogenic than those produced by canonical mutations. Thus, both renal-associated mutants may represent less aggressive forms of AGel amyloidosis compared to D187N, due to their reduced propensity of their fragments to form amyloid.

### G4G5 variants, a novel hotspot of amyloidogenicity

For a long time, the furin-dependent pathway was considered the only triggering mechanism for forming GSN pathological deposition in AGel. Since 2018, several new mutations falling far from the G2 domain have been discovered, suggesting a possible alternative aggregation mechanism (Fig. 4 and Table 2). Five novel mutations lie at the interface between domains G4 and G5, namely A551P, Y447H/N, E553K, M517R, and W466R. Patients carrying G4-G5-linked mutations present heterogeneous clinical pictures, including some characteristic features of AGel amyloidosis such as corneal lattice dystrophy and neurological symptoms.

A single patient carrying the A551P mutation [84] has been described so far, and his clinical features are further complicated by the concomitant presence of mutated (V122I) ATTR deposits. However, the two causative proteins seem to deposit in different organs with aggregated GSN found solely in aspirated abdominal fat and biopsy of the gastrointestinal tract. No typical signs of AGel amyloidosis were reported. Overall, A551P substitution seems to cause an asymptomatic amyloidosis or a very mild form of AGel at the latest. Mild is also the phenotype linked to the Y447H/N mutation. Of the two Y744H carriers described [85], the father was reported to be asymptomatic, whereas the daughter only suffered from mild carpal tunnel syndrome along with amyloid deposits in breast tissue. Y447 substitution in Asn as well does not show any classical AGel features. The amyloidosis results by the dual aggregation of the GSN and wild type ATTR proteins in the heart but in two different anatomic compartments (vasculature for GSN and interstitium for ATTR [86]). The E553K and M517R variants are associated with more severe phenotypes similar to those caused by D187N-mutated GSN, with the E553K carrying patient also exhibiting cardiac involvement [87]. The W466R variant [88] was identified in the G4 domain, causing the formation of corneal amyloid deposits. Immunohistochemical studies demonstrate the presence of the C-terminal portion of GSN, once again suggesting the deposition of the protein in its full-length form.

Mass spectrometry of amyloid deposits is a crucial analysis linking clinical and molecular phenotypes of novel forms of AGel amyloidosis. Unfortunately, this diagnostic test is not always performed, and the complete data is rarely presented in clinical reports. Mass spectrometric data for a patient with the A551P mutation are available [84]. As seen in the case of G167R [81], it identified portions spanning the entire protein rather than the expected 8 and 5 kDa fragments, suggesting a potential alternative mechanism of aggregation. A similar analysis was also conducted on the amyloid deposits from patients with Y447H/N [85], once again revealing several regions of the full-length protein.

As expected, unlike the variants in the G2 domain, none of the M517R, A551P, and E553K novel variants were susceptible to furin cleavage [47] (Table 3). The full-length proteins’ structure and stability are affected by the substitutions to a different extent, with A551P having the mildest effect, and M517R and E553K being strongly destabilized (Table 3). In particular, E553K undergoes denaturation already at physiological temperature. Moreover, in contrast with all the other variants, E553K can sever actin’s filaments even without calcium, suggesting a compromised compactness of the inactive state of GSN. Analysis of the crystallographic and simulated models suggests three different effects of the substitutions in the region of interest. They all lay within or near the two parallel β-strands (residues 517–520 and 549–553), constituting the interface between G4 and G5 domains. The alanine substitution with proline at position 551, known as a β-breaker residue, causes a distortion of the β-strand and the loss of one H-bond with M517. The glutamic acid to lysine substitution at position 553 causes local rearrangements due to charge repulsion. As for M517R, introducing a charged and bulky residue destroys the cluster of interaction with the neighboring hydrophobic residues within the sulphur-aromatic (S-π) motif, [89] which appears to play a significant role in the stability of the G4 domain. Molecular dynamic simulations hint at an acquired increased dynamicity in the G4:G5 regions of all three variants to a different extent, with M517R having the most severe phenotype.

Y447H/N and W466R substitutions in the G4 domain are the most recently identified variants; no in vitro studies of these variants are currently available [85, 88]. However, both substituted residues fall in the same S-π motif of which M517R is part (Fig. 3, G4:G5 inset), suggesting that these substitutions may have a similar impact on the biophysical features of the protein. Adding a negative or positive charge could lead to a distortion of the close secondary structures, thus destabilizing the interface between these two domains, triggering the same aggregation mechanism of the above-mentioned variants.

As a result of the instability of the interface between G4:G5, the three characterized variants A551P, E553K, and M517R are endowed with the tendency to aggregate as amyloid deposits, even in their unproteolysed full-length form. In contrast to what happens with the G2 variants, incubating the mutated proteins A551P, E553K, and M517R in the absence of calcium leads to an increase in the thioflavin T fluorescence over time. This suggests that the acquired flexibility of the mutants exposes one or more unidentified aggregation-prone sequences that are otherwise buried in the WT protein [47].

### Frame-shift mutations with an Alzheimer’s disease-like phenotype

To date, three frameshift (FS) mutations have been associated to an Alzheimer’s disease-like amyloidosis; a phenotype never observed for any AGel missense mutations reported so far. These FS variants are associated with cerebral lesions and do not present the clinical features typically observed in AGel amyloidosis [90–92]. When translated, the frameshift variants result in truncated proteins with altered sequences: the c.100dupG produces an 11 kDa fragment, A34fs; the c.8_35del a 10 kDa fragment, P3fs; and the mutant c.1036delA a 53 kDa protein, K346fs. The translated A34fs fragment accumulates in the brain, leading to the development of multiple cerebral cavernous malformations and associated hemorrhagic lesions [90]. The K346fs and P3fs mutations were identified during a genetic screening of patients with diagnosed with Alzheimer’s disease, aimed at detecting amyloidogenic proteins, such as GSN, cystatin C, transthyretin, and integral membrane protein 2B. The GSN K346fs and P3fs frameshifts were found in 5 Alzheimer’s disease ’s patients, however no known pathogenic protein deposition was observed, thus their role in the pathogenesis of Alzheimer’s disease is yet unclear. Both variants are associated with cognitive decline and psychiatric symptoms and do not exhibit systemic AGel features. P3fs is characterized by a late-onset, more severe phenotype aggravated by personality changes and multisystem involvement affecting the eyes, skin, and thyroid. Patient carrying the K346fs mutation exhibited brain atrophy and Aβ protein deposition was observed in bilateral cerebral cortex and subcortical nuclei [92].

**Fig. 5:**
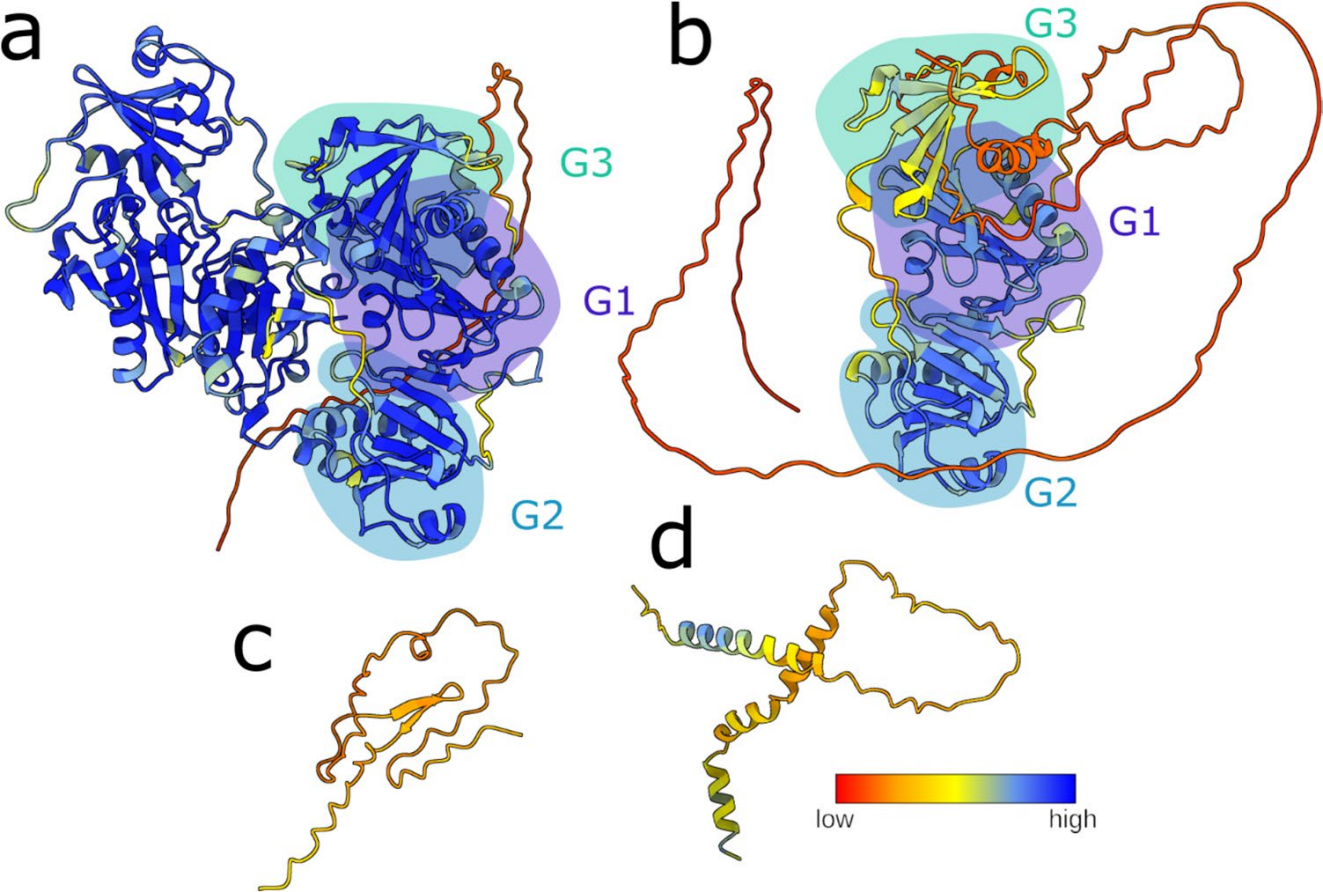
Alphafold structure prediction of the GSN frameshift mutants. The prediction was calculated using the gene sequence NM_000177.5 as a reference, thus including the peptide signal as it also hosts pathogenic mutations. The color key represents the structure prediction confidence. **a** Structure prediction of the full-length GSN, the red tail visible is the signal peptide. **b** Frameshift mutant c1036delA, which has no impact on domains G1 and G2, whereas truncates G3 which retains some structure according to the prediction. The same orientation as in panel a is shown. **c** Prediction for mutant c8_35del. Two β strands with low confidence are visible. **d** Prediction for the mutant c100dupG shows some α-helices content

A biochemical characterization of the three frameshift variants is missing, and we here used Alphafold 3 [93] for some molecular insights (Fig. 5). The two shorter variants are predicted to be disordered, while the P346fs mutation may not significantly affect the first two domains and a portion of G3, which might still be structured. However, a long stretch of the P346fs protein is predicted to be disordered with hydrophobic, potentially aggregation-prone, sequences exposed to the solvent. The mutants’ propensity to aggregate was also investigated in silico using AMYLPRED2 [94]. Compared to the full-length WT GSN, no new amyloidogenic sequences were found in the K346fs mutant. However, sequences with a significant consensus have been detected in both short mutants, indicating that they may be amyloidogenic.

Several studies have highlighted a correlation between GSN and Alzheimer’s disease, demonstrating that GSN binds to the Aβ peptide, hampers its aggregation, and delays disease progression by protecting cells from apoptosis and radical oxygen species. It has also been suggested that decreased plasma GSN levels could serve as a biomarker for Alzheimer's disease and its progression. In the context of Alzheimer's disease and Aβ aggregation, the truncated frameshift mutant proteins may not be translated, and the resulting loss of function could impair GSN-mediated cellular protection [92].

### Other mutations and a sporadic form of AGel

Two recently discovered mutations, P432R and L538P [95, 96], remain unclassified as they are located outside the classical hotspots, the G2 Ca^2+^-binding site and at the interface between domains G4 and G5. Neither P432R in the G4 domain nor L538P in the G5 domain has been characterized in vitro. Although P432 is situated at the interface between G3 and G4, it does not directly participate in the interactions between these two domains. Instead, it is exposed to the surface of the protein. The substitution of proline with arginine not only increases the size and bulk of the side chain but may also disrupt the secondary structure of the α-helix and alter the charge of the protein's electrostatic interactions at the interface. The patient with the P432R mutation, a male of African descent, presented with an atypical clinical profile that included fevers, skin rash, polyneuropathy, and anemia [95].

The patients in whom GSN carries the L538P substitution exhibit reticular corneal dystrophy and carpal tunnel syndrome, but no relevant dermatological symptoms [96]. L538 lays in the middle of the β-sheet constituting the G5 domain and, the substitution with a proline, with its rigid, cyclic structure can introduce a *kink* in the polypeptide chain and disrupts the secondary structure of the β-sheet, where leucine might stabilize the overall fold. The change can then interfere with the overall protein flexibility.

Although we believe AGel amyloidosis should be considered a hereditary disease, there is a single report of an acquired form of the disease in which amyloid deposits of the WT protein were found surrounding a pituicytoma, a brain tumor originating from the pituitary gland [97]. In this Chinese patient, the deposition of GSN forms a solid sellar mass with suprasellar extension. Sequencing of the *gsn* gene failed to identify any mutation but typing of the amyloidosis was performed by the robust proteomic analysis of the ex vivo aggregates. Moreover, such analysis identified GSN fragments not limited to 5 and 8 kDa fragments but spanning the whole sequence, similarly to aforementioned variants. Under harsh conditions, such as acidic pH, WT GSN shows mild amyloidogenic potential ([78] and unpublished data from authors). Additionally, GSN is overexpressed in few cancers [98–102] and its fragmentation observed in melanomas [103, 104]. This raises a compelling, yet untested, hypothesis: that abnormal accumulation of the WT protein, even in the absence of mutations, might be sufficient to drive its aggregation under certain pathological conditions.

At the time of writing, two novel possibly-pathogenic variants of GSN have been described: G248S (c.823G > A, p.Gly275Ser), [105] and D737H (author numbering scheme: c.2245G > C, p.Asp749His) [106]. In both clinical reports, AGel is discussed and in the latter even considered for the diagnosis, but patients are very young compared to standard onset of Agel amyloidosis and no amyloid deposits have been observed in any tissue. Although mutations in GSN are mainly associated with the amyloidosis, due to the multifunctional role of the protein may lead to other diseases. Indeed, the patient associated with G248S only presented sever pruritis and burning sensations, whereas the D737H carrier was mainly affected by nephropathy and thrombotic microangiopathy.

## Investigation on therapeutic approaches

Currently, patient management does not address the underlying biochemical mechanisms driving the pathology but instead focuses primarily on symptom relief through multiple surgeries and invasive interventions. For this reason, the search for therapeutic compounds capable of disrupting the biochemical pathway leading to misfolded GSN deposition should be considered a priority.

**Fig. 6:**
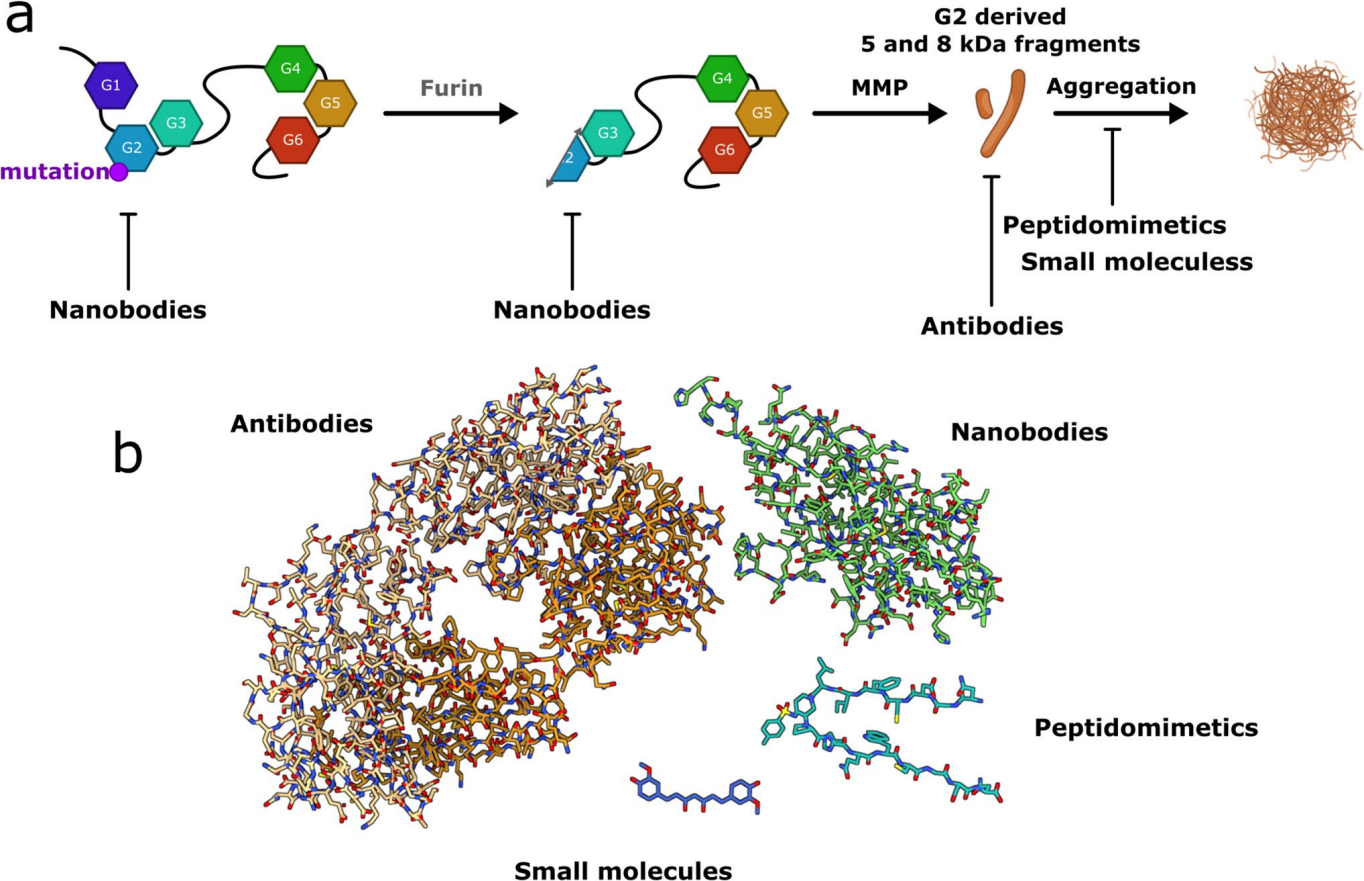
Targets and chemical nature of investigational therapeutics. Several classes of molecules have been investigated to target different steps of the furin-dependent pathogenic pathway. **a** Nanobodies (Nbs, single-domain antibodies) raised against the G2 domain stabilize the native fold of gelsolin and prevent aberrant furin proteolysis. Other Nbs targeting the C68 fragment protect against the subsequent proteolytic cleavage. Fragment antigen-binding antibodies (Fabs, composed of one constant and one variable domain of each of the heavy and the light chain) have been identified against the aggregation-prone 5 and 8 kDa gelsolin fragments and effectively inhibit their self-aggregation. **b** Similar anti-aggregating activity has also been reported for several small natural compounds, including plant-derived cytokinins and polyphenols such as curcumin, whose structure is shown as a representative example. In addition, rationally designed gelsolin-specific peptidomimetics have been shown to efficiently inhibit aggregation

The two defining features of all AGel forms are the accumulation of amyloidogenic deposits in various tissues and the lack of pharmacological treatments capable of blocking or slowing disease progression. Several strategies have been explored to inhibit amyloid formation, including rescuing mutant GSN misfolding using structural chaperones, reducing the production of amyloid precursor proteins/fragments with anti-aggregants, or employing disaggregating molecules to promote the clearance of pre-existing aggregates (Fig. 6). Conversely, inhibiting the proteases involved (furin and one or more matrix metalloproteinases) is not a viable option due to their essential physiological roles in human cells and the potential for significant side effects [107, 108].

Most investigational therapeutic approaches target the aggregation of amyloidogenic fragments; however, these strategies may be ineffective for variants characterized by proteolysis-independent mechanisms. Other approaches focus on stabilizing the protein to prevent misfolding and proteolytic events, which may also be variant-specific. Although stabilizing the protein in its monomer conformation can reduce the formation of new disease-relevant structures [12], this approach might increase intracellular protein concentration, impairing cellular homeostasis. In addition, other processes, such as those regulated by molecular chaperones and autophagy, which help in preventing aggregation, may be affected. The clearance of accumulated monomeric protein may be compromised, allowing the formation of unstable, toxic oligomers. This scenario is much more complicated in the case of GNS, whose aggregation process has not been thoroughly characterized, and proteolytic processes on different domains can form amyloidogenic peptides, rendering the design of anti-aggregation compounds very challenging.

The fibrillogenic process — *i.e.*, the aggregation of misfolded proteins into oligomeric conformations, which subsequently form higher-order fibrils and plaques— has been extensively studied to prevent deposit formation. Historically, amyloid plaques were considered the primary drivers of pathogenesis. However, over time, it has become evident that the main pathological agents are small, soluble, and cytotoxic oligomers capable of initiating degenerative cascades, rather than mature fibrils, which are also toxic but to a lesser extent. Small molecules, short β-sheet breaker peptides/peptidomimetics, and antibodies targeting amyloid fibrils and amyloid-like aggregates can interfere with the amyloid aggregation process. In this context, an effective anti-aggregation compound should not selectively inhibit fibril elongation while allowing the accumulation of highly toxic soluble oligomers [109–112].

The approaches aimed at reducing the GSN amyloid burden are here reviewed, classifying them according to the nature of the molecules involved, including small molecules, peptidomimetics, and biologics, such as anti- and nano-bodies (Fig. 6).

### Small molecules with anti-aggregating activity

Curcumin and emetine, two bioactive molecules selected through virtual screening, exhibited distinct effects on GSN aggregation [113]. Curcumin promoted aggregation into higher-order structures, leading to the formation of larger fibrils, whereas emetine blocked aggregation at the oligomeric stage, preventing the formation of mature fibrils.

Since GSN assembly is initiated by stacking interactions between F183 and F189, molecular dynamics (MD) simulations indicate that curcumin can establish a strong triplanar stacking interaction with the protein. Moreover, curcumin is planar and symmetrical enough to interact with two GSN molecules simultaneously. This spatial arrangement results in extended hydrophobic surfaces that accelerate multipolar GSN assembly into fibrils. Conversely, emetine has been found to form hydrogen-bonding interactions with the terminal S182 of GSN through its bulky isoquinoline ring. As a result, only one end of GSN remains accessible for assembly, leading to the formation of oligomers whose further extension into fibrillar structures is sterically hindered.

The same authors later proposed encapsulating curcumin and emetine in poly(lactic-co-glycolic) acid nanoparticles to enhance their effectiveness and bioavailability [114]. Nanoparticles enabled lower compound concentrations to achieve a faster and more potent modulatory effect. When encapsulated in nanoparticles, curcumin accelerated fibrillation, while emetine halted aggregation and induced the formation of non-fibrillar aggregates. The aggregates formed in the presence of nanoparticles exhibited reduced toxicity compared to untreated GSN fibrils, as demonstrated by cytoxicity studies on the human SH-SY5Yneuroblastome cells.

More recently, plant-derived cytokinins, such as kinetin and trans-zeatin, have emerged as a new class of small-molecule modulators of GSN aggregation [115]. Unlike curcumin, which enhances fibril formation, or emetine, which stabilizes oligomeric species, cytokinins primarily act as disaggregation promoters. Biophysical and mass spectrometry studies demonstrated that these compounds form non-covalent 1:1 complexes with GSN-derived peptides and their pathogenic variants, inhibiting fibril formation and partially reversing pre-formed aggregates. Molecular docking analyses suggested that cytokinin activity arises from a combination of hydrogen bonding and aromatic π–π interactions between the adenine core and key residues within the amyloidogenic region, including phenylalanine and tyrosine. Notably, trans-zeatin displayed greater efficacy than kinetin and reduced aggregate-associated cytotoxicity, highlighting the importance of the N6-substituent in modulating inhibitory potency.

Two other small molecules, the methylene blue and the polyphenol (-)-epigallocatechin gallate, known to be active against tau and Aβ aggregation, were evaluated for their ability to inhibit the aggregation of various peptides containing the GSN amyloidogenic sequence CFILDL [77]. The study demonstrated that both compounds act as dual-function inhibitors, preventing the aggregation of GSN peptides and promoting the disaggregation of pre-formed fibrils. Notably, both inhibitors effectively reduced the aggregation, with IC_50_ values ranging from 2 to 13 µM. However, their precise mode of action was not fully elucidated. The authors proposed that their inhibitory mechanism involves both the oxidation of cysteine residues within GSN amyloidogenic peptides and hydrophobic or π–π interactions with aromatic amino acids, such as phenylalanine 183, present in the hydrophobic CFILDL sequence.

The toxicity associated with amyloid aggregation can also be countered by accelerating the process. To this end, oxidized phospholipid 1-palmitoyl-2-(90-oxononanoyl)-sn-glycero-3-phosphocholine (PoxnoPC) has been reported to enhance GSN aggregation in a concentration-dependent manner [116]. Since nucleation is affected only when the concentration of PoxnoPC promotes the formation of micelles, it is suggested that the water-membrane interface can play a role in GSN aggregation, similar to other amyloidogenic systems.

### Peptidomimetics

Our group has recently developed anti-aggregating peptidomimetics following a previously applied strategy to two other amyloidogenic proteins, Aβ and human islet amyloid polypeptide aggregation [117–119]. A set of peptide mimics containing a piperidine-pyrrolidine scaffold proved capable of interfering with the aggregation of the 5 and 8 kDa fragments produced by the furin-dependent proteolysis of the G2-linked variants, which are the most common and widespread [79]. These molecules were designed based on the sequence of the GSN amyloidogenic core (residues 182-SFNNGDCFILD-192), which triggers the aggregation of the fragments, and the sequence of the flanking β-strand (194-GNNIHQWCGSN-204). These peptidomimetics interfere with the aggregation of the amyloidogenic core sequence and the isolated D187N-mutated G2 domain at promising concentrations by acting as molecular tweezers that sequester the amyloidogenic core. These compounds represent an innovative strategy to interfere with GSN aggregation by mimicking the interactions between peptide sequences and preventing fibril formation. Furthermore, once the triggering amyloidogenic sequence is known, this approach could work for any GSN amyloidogenic proteins, such as the variants at the interface between the G4 and G5 domains.

### Biologics

Several *Camelidae* species express a unique class of antibodies composed solely of heavy chains. These antibodies can be engineered into nanobodies (Nbs), *i.e.*, single heavy-chain variable domains, which are fully functional even in the absence of a light chain. These Nbs represent the smallest intact antigen-binding fragment of a heavy-chain immunoglobulin and are versatile tools for fundamental research, diagnosis and treatment [120, 121].

A library of GSN-specific Nbs was developed by injecting human recombinant GSN into a llama, each binding to different epitopes on the protein surface [122]. Furthermore, the Nbs can distinguish between the various conformational states of GSN, including its free form, the calcium-activated state, and the actin-bound configuration. This specificity makes them valuable tools for addressing key biological questions and elucidating the biochemical mechanisms underlying structural transitions and interactions within cellular contexts.

One of these Nbs, Nb11, recognizes the epitope in the G2 domain (G137–L247). The crystal structures of G2 WT and D187N mutant in complex with Nb11 (PDB codes 4S10 and 6H1F, respectively) show that Nb11 binds an extended area including the β5-loop-α2 region (residues 230–234, 238–245) and the loop-β4 stretch (residues 193–198). Despite being distant from the furin binding site, Nb11 protects both the full-length GSN and isolated G2 domain from furin proteolysis in vitro and in vivo assays, being active in the trans-Golgi network of human cells and transgenic GSN amyloidosis/Nb double-positive mice [123, 124]. Structural analysis of crystallographic B-factors and MD simulations of Nb11 with the G2 pathological variants show a gain in structural stabilization propagating through the C-terminus and the α2 helix of the variants’ structures. The characterization of Nb11 shows that it works as a pharmacological chaperone, thermodynamically stabilizing all the G2 variants, reverting the misfolding effect of the substitutions and counteracting their proteotoxicity both in vitro and in vivo [71, 124, 125].

The same authors generated a second generation of GSN Nbs (FAF-Nb) by immunizing llamas with the 8 kDa fragment and the G2 domain, harboring the substitution D187N [123]. FAF-Nb specifically binds the mutated C68 GSN fragment by recognizing an epitope unique to mutant GSN and more accessible in the C68 degradation product. These Nbs shield the C68 amyloid precursor protein from proteolysis by membrane-type 1 matrix metalloproteinase, thus preventing the formation of the 8 kDa peptide [123]. A very thorough summary on the two generations of Nbs was written by Verhelle and Gettemans in 2016 [126]. A bispecific Nb was then developed to simultaneously shield mutant plasma GSN from intracellular furin and extracellular membrane-type 1 matrix metalloproteinase activity [127].

In addition to acting as chaperones, Nbs and antibodies can counteract amyloidogenic deposition by targeting the aberrant amyloidogenic 8 kDa fragment (A173-A242). Leimu et al. [128] generated a panel of high-affinity antigen-binding antibody fragments (Fab) by phage display that recognize different epitopes of the 8 kDa fragments in the amyloidogenic sequence and the flanking regions. Three of these Fabs bind and inhibit the amyloid formation in vitro at a stoichiometric ratio 1:1 [128].

Their high specificity, stability, versatility, and ease of engineering to optimize pharmacokinetic properties provide antibodies, Fabs, and Nbs a distinct advantage in diagnostic or clinical applications and targeted therapeutic approaches. They can serve as noninvasive imaging probes to identify diseased tissues and circulating pathological proteins. Notable examples of antibodies currently in clinical trials include aducanumab and lecanemab, which target Aβ in patients with Alzheimer’s disease [129]. However, the large size of antibodies and Nbs may hinder their ability to penetrate cells effectively. This indicates that developing smaller drug-like molecules or peptides that retain nanobodies' protective, or chaperone-like functions could provide a more efficient therapeutic alternative.

## Pre-clinical models of AGel

In vitro and in vivo models replicating the clinical features seen in patients are essential for clarifying the mechanisms underlying AGel and conducting preclinical pharmacological studies. However, similar to other rare systemic amyloidoses, limited efforts have been made to develop affordable models for AGel.

To investigate whether the mutations associated with FAF-type AGel can be processed in vitro to produce amyloidogenic fragments, monkey kidney fibroblast-like COS-1 cells were engineered to express D187N- or D187Y-mutated human GSN [62]. Mutant forms were homogeneously distributed throughout the cytoplasm and were subjected to abnormal proteolysis in vitro, resulting in the secretion of an aberrant 68 kDa carboxyterminal fragment into the culture medium. This resulted in intracellular partially aggregated deposits. No additional in vitro models and no induced pluripotent stem cells have been developed to create cellular models of AGel variants relevant to humans [130]. More recently, a pharmacological study investigated the protective effect of curcumin and emetine encapsulated into poly lactic-co-glycolic acid (PLGA) nanoparticles against the toxicity induced in human SH-SY5Y neuroblastoma cells by fibrillar aggregates of the recombinant 8 kDa GSN amyloidogenic peptide [114]. The 24-h exposure to aggregates reduced the cell viability by about 60%. Nanoparticles containing curcumin and emetine, which are effective in inhibiting GSN aggregation, reduced this toxicity, indicating that proteotoxicity is linked to the fibrillation process.

The availability of in vivo models is restricted, too. The only transgenic animal currently available is a mouse developed to study FAF-type AGel, in which human D187N-mutated GSN was expressed under the control of a muscle-specific promoter. This approach enabled an accurate reproduction of the proteolytic cascade leading to the formation of the 8 and 5 kDa amyloidogenic fragments, reflecting the pathological process observed in FAF patients [114]. One of the most significant aspects of this model is the localization of amyloid aggregation, which, despite the presence of full-length protein and the C68 fragment in the blood, occurs exclusively in muscle tissues synthesizing the mutant GSN. Furthermore, mice homozygous for the D187N mutation exhibited a progressive loss of muscle strength and a decline in cellular proteostasis, accompanied by intracellular accumulation of amyloidogenic proteins, phenomena also seen in sporadic inclusion body myopathy (sIBM) in humans [131]. Despite its usefulness, the model has some limitations. Although it successfully reproduces the proteolytic cascade and local amyloid deposition, it remains unclear whether it fully replicates all clinical manifestations of FAF, such as peripheral neuropathy, *cutis laxa*, or corneal dystrophy observed in patients [132]. Utilizing a muscle-specific promoter restricts the synthesis of the mutant protein to this tissue. In contrast, in human pathology, the mutant GSN is expressed in several organs [133], which may diminish the model's ability to represent the disease's multisystem manifestations. Additionally, species-specific differences between mice and humans could affect disease progression and response to potential treatments. GSN metabolism and proteostasis regulation may not perfectly overlap between the two species. Despite these concerns, this transgenic mouse model serves as a crucial tool for studying the pathogenetic mechanisms of FAF and evaluating potential therapeutic strategies, including inhibition of furin and membrane-type 1 matrix metalloproteinase, which trigger GSN cleavage, utilization of proteostasis regulators, and antagonism of interactions between amyloidogenic fragments and glycosaminoglycans.

No new transgenic strains have been developed as AGel models, and alternative methods have been used to study the toxicity of the various GSN variants.

*Caenorhabditis elegans* has recently been utilized as an animal model to investigate the proteotoxic effects of WT and mutated GSN. This nematode is commonly used in developmental biology and genetics due to its transparency, short life cycle, and ease of genetic manipulation. Furthermore, it serves as a well-established experimental system for studying amyloidogenic diseases because of its capacity to recognize the proteotoxic assemblies of misfolded proteins [134–136]. This recognition is indicated by a dysfunction of the feeding behavior, particularly the pharyngeal contraction, measurable by the frequency of pharyngeal pumping [137].

Studies were performed to evaluate whether the presence of mutations in the G2 domain of GSN can affect its proteotoxic effect. The G2-mutated variants significantly reduced pharyngeal contraction frequency, with more pronounced effects for the N184K mutation than for the G167R and D187N variants [71]. Pharyngeal impairment was persistent over time, suggesting lasting structural damage. Furthermore, the toxicity of the variants was dose-dependent and correlated with the conformational state of the protein: the unstructured form of G2 showed a more remarkable ability to inhibit pharyngeal function than the correctly folded form. The use of Nb11, designed to stabilize the structure of the protein, neutralized the toxicity of the G2 variants in nematodes, restoring normal pharyngeal function in both the short and long term [71].

The proteotoxicity of the new G4:G5 variants of amyloidogenic GSN was also investigated [47] by treating worms with WT GSN and mutant variants (D187N, A551P, E553K, and M517R) in the presence or absence of calcium, and after prolonged incubations under conditions mimicking the aggregation process. The results showed a clear correlation between protein destabilization and toxicity, with a reduction in pharyngeal function proportional to the ability of the variants to aggregate. In particular, the M517R mutation was the most toxic, inducing a reduction in pharyngeal function of up to 37% in the first hours after exposure. Toxicity increased over time for some variants, suggesting a role of structural destabilization in the progression of cell damage. Additional experiments showed that proteotoxicity was dependent on the conformational state of the proteins: treatment with high temperatures abolished the toxic effect, indicating that recognition by *C. elegans* depends on the three-dimensional structure of the proteins rather than their mere presence. These results suggest that the aggregation of the G4:G5 variants may follow an alternative, proteolysis-independent mechanism to the classical GSN variants, opening new perspectives in the study of pathogenesis and the search for potential therapeutic targets.

*C. elegans* was employed in a subsequent study as a rapid and inexpensive system for preclinically evaluating the potential activity of compounds to protect against GSN toxicity. This would provide a helpful platform for testing new pharmacological strategies against AGel, particularly in the absence of animal models. Specifically, the ability of previously described peptidomimetics to counteract GSN proteotoxicity was evaluated [79]. Three peptidomimetics, LB-5, LB-6, and LB-7, were tested for their effectiveness in countering the aggregation and toxicity of the GSN amyloidogenic core (GAC), identified in systematic studies as spanning residues 182 to 192. LB-5 and LB-6 efficiently inhibited the aggregation of the GAC at sub-stoichiometric concentrations and were also effective in vivo in *C. elegans*, counteracting proteotoxicity. In contrast, LB-7 failed to alleviate the harmful effects of the aggregates and worsened the nematode's condition. These data indicate that LB-5 and LB-6 peptides, although at micromolar concentrations, counteract the toxicity of either the fibrils or some soluble species in equilibrium with the amyloids.

## Future trends

In the last decade, AGel amyloidosis has evolved from a disease linked to only a few mutations in specific areas of northern Europe to a condition characterized by diverse clinical symptoms and associated with several mutations that can lead to amino acid substitutions in various protein domains. Although it remains a very rare disease, AGel has thus transformed into an amyloidosis that may appear in multiple regions around the world, making diagnosis challenging were it not for the advancements in recent years in next-generation sequencing (including whole exome sequencing and whole genome sequencing). Identifying the underlying triggers and mechanisms related to new mutations underscores the vital role of laser microdissection and mass spectrometry analysis of amyloid deposits in patients with Agel [27–29]. This approach combines the precision of laser microdissection, which enables the isolation of specific tissue regions containing amyloid plaques, with the sensitivity of mass spectrometry to detect and characterize the proteins and peptides present in these deposits. This will be relevant for examining the molecular composition of amyloid, gaining insights into the pathophysiology of the specific amyloid-related variant, and also contributing to identifying biomarkers useful for the rapid diagnosis of AGel.

As is often the case with most systemic amyloidoses, even for AGel, the diagnosis frequently arrives late, after physicians have ruled out a series of more common pathologies. Timely diagnosis is complicated by the lack of a clear description of the various symptoms associated with the different AGel variants linked to specific mutations. This is partly due to the small number of patients with the same mutation.

To further complicate the situation, there is a lack of in vitro preclinical models, including a shortage of relevant 2D and 3D cellular models and organoids expressing the mutated forms of the protein, which can significantly contribute to elucidating the mechanisms underlying proteotoxicity and identifying druggable targets with high affinity. Together with the lack of transgenic animal models, both invertebrates and vertebrates, this hinders the development of effective pharmacological treatments capable of reducing aggregate toxicity. Furthermore, the lack of animal models impedes the evaluation of the drug's toxicity and safety profile, making it difficult to predict how effectively its protective effects will translate to Agel patients.

Recently, treatments based on small interfering RNA and antisense oligonucleotides have been proposed for variant ATTR polyneuropathy and have been shown to produce symptom relief, functional improvement, and prolong patients’ survival [138]. However, these therapies require long-term, repeated administration to maintain TTR knockdown. To overcome this problem, new clinical studies are ongoing to evaluate the efficacy of CRISPR–Cas9 in silencing the TTR gene [139]. The lack of in vivo models resembling GSN aggregation and proteotoxicity mechanisms remains a central limitation in assessing whether molecular-based and gene-editing strategies may be effective for Agel.

Even the mouse model that expresses D187N-mutated GSN appears to be underutilized. No further works have been published since 2020 utilizing it for in-depth studies on the mechanisms underlying the pathology or for preclinical studies. Indeed, the lack of funding for studying a rare disease like AGel does not facilitate this type of research, which requires substantial investments and extended timeframes to yield results. Developing a simpler and less expensive model could greatly help in this context.

Our group is making significant efforts to develop a new in vivo AGel animal model using *C. elegans* as a complementary system for studying GSN aggregation and toxicity. This work aims to bridge the gap between the molecular events observed in vitro and patients' clinical and pathological changes. Utilizing this invertebrate nematode is cost-effective and avoids the legislative limitations and ethical concerns associated with mammals. We rely on the fact that the genome of *C. elegans* encodes three GSN-related proteins: flightless-1, villin-like protein, and gelsolin-1 (GSNL-1) [140]. Notably, gene expression analysis has shown that GSNL-1 mRNA is highly enriched in the body muscles of nematodes, suggesting a specific role in regulating the actin cytoskeleton structure in striated muscle. Moreover, the *gsnl-1* gene encodes an unconventional protein with four G domains. Importantly, the G1 and G2 domains of GSNL-1 have sequences similar to their human counterparts and exhibit comparable calcium-dependent activity on actin filaments [141]. *C. elegans* also expresses an ortholog of human furin, KPC-1, which possesses serine-type endopeptidase and signaling receptor binding activities, and is mainly involved in several neuronal processes and protein processing [142]. With these premises, we have recently generated two new transgenic *C. elegans* strains expressing the WT or D187N-mutated, and secreted mature form of human GSN in the body-wall muscle cells. These strains, which still require thorough genotypic and phenotypic characterization, will be critical in clarifying whether the worm’s furin can process the human protein to form amyloidogenic fragments and understanding the relationship between their aggregation, localization, and proteotoxic effects. This new data could pave the way for developing additional animal models expressing other mutated forms of human GSN to elucidate how each mutation is associated with specific mechanisms.

Another critical point, especially for patients, is the development of new therapeutic approaches aimed not only at containing the symptoms of the disease but also at curing it. In this regard, providing a better description of the physiological activity of GSN and a more detailed structural characterization of the proteins in its various variants is a priority for identifying druggable targets.

## Data Availability

Not applicable.
